# Paraganglioma of the urinary bladder initially diagnosed as gastrointestinal stromal tumor requiring combined resection of the rectum: a case report

**DOI:** 10.1186/s12957-022-02662-7

**Published:** 2022-06-08

**Authors:** Natsumi Matsuzawa, Takeshi Nishikawa, Riki Ohno, Masaharu Inoue, Yu Nishimura, Tomomi Okamoto, Takao Shimizu, Takahide Shinagawa, Yusuke Nishizawa, Shinsuke Kazama

**Affiliations:** 1 Department of Gastroenterological Surgery, Saitama Cancer Center, Saitama, 362-0806 Japan; 2 Department of Urology, Saitama Cancer Center, Saitama, Japan; 3 Department of Pathology, Saitama Cancer Center, Saitama, Japan

**Keywords:** Paraganglioma of the urinary bladder, Large pelvic tumor, Hypertension

## Abstract

**Background:**

Paraganglioma of the urinary bladder (Pub) is rare and presents with clinical symptoms caused by catecholamine production and release. The typical symptoms of Pub are hypertension, macroscopic hematuria, and a hypertensive crisis during micturition. The average size of detected Pubs is approximately 3 cm. Herein, we report a case of a large Pub in which the symptoms were masked by oral medication, precise preoperative diagnosis was difficult, and intraoperative confirmation of tumoral adhesion to the rectum resulted in hypertensive attacks during surgery.

**Case presentation:**

A 64-year-old Japanese male with a history of hypertension and arrhythmia controlled with oral medication presented with a large tumor in the pelvic region, detected on examination for weight loss, with no clinical symptoms. Computed tomography and magnetic resonance imaging revealed a tumor measuring 77 mm in diameter in the posterior wall of the urinary bladder. The border with the rectum was unclear, and the tumor showed heterogeneous enhancement in the solid part with an enhancing hypodense lesion. Cystoscopy revealed compression of the bladder trigone by external masses; however, no tumor was visible in the lumen. Endoscopic ultrasonography-guided fine-needle aspiration revealed CD34-positive spindle-shaped cells in the fibrous tissue, suggestive of a mesenchymal neoplasm. The tumor was suspected to be a gastrointestinal stromal tumor, and surgery was performed. After laparotomy, we suspected that the tumor had invaded the rectum, and total cystectomy and anterior resection of the rectum were performed. Histologically, the tumor cells had granular or clear amphophilic cytoplasm with an oval nucleus and nests of cells delimited by connective tissue and vascular septations. Immunohistochemically, the tumor was positive for chromogranin A, CD56, and synaptophysin, and a diagnosis of paraganglioma of the urinary bladder was confirmed. There was no tumor recurrence at the 7-month follow-up.

**Conclusion:**

This case highlights the importance of careful examination of pelvic tumors, including endocrine testing, for detecting paraganglioma of the urinary bladder in patients with a history of hypertension or arrhythmia.

## Background

Pheochromocytoma (PCC) is a catecholamine-producing tumor originating from chromaffin cells derived from the adrenal medulla. Tumors derived from extra-adrenal ganglia are called paragangliomas (PGLs) and occur in 10–15% of all PCC cases [1]. PGL of the urinary bladder (Pub), first described by Zimmerman in 1953 [2], is rare, occurring in 10% [1] and < 0.06% [3] of all PGL and bladder tumor cases, respectively. Symptoms of Pub are caused by catecholamine production and release [1]. The typical symptoms are hypertension, macroscopic hematuria, and hypertensive crisis during micturition, along with headache, blurred vision, and palpitations [3]. However, some cases without typical symptoms have been found incidentally, making preoperative diagnosis difficult. Herein, we report a case of a large Pub in which a precise preoperative diagnosis and intraoperative confirmation of tumor adhesion to the rectum were difficult, resulting in hypertensive attacks during surgery.

## Case presentation

A 64-year-old male with a history of hypertension, tachyarrhythmia, and diabetes presented with a large bladder tumor incidentally detected on examination for weight loss. The patient was on antihypertensive, pulse stabilizer, and antidiabetic medications, and his blood pressure was 121/67 mm Hg. The patient’s abdomen was soft and flat on physical examination, and the tumor was not palpable on digital rectal examination. Laboratory examinations revealed elevated carcinoembryonic antigen (6.3 ng/mL) and α-fetoprotein (14.9 ng/mL).

**Fig. 1:**
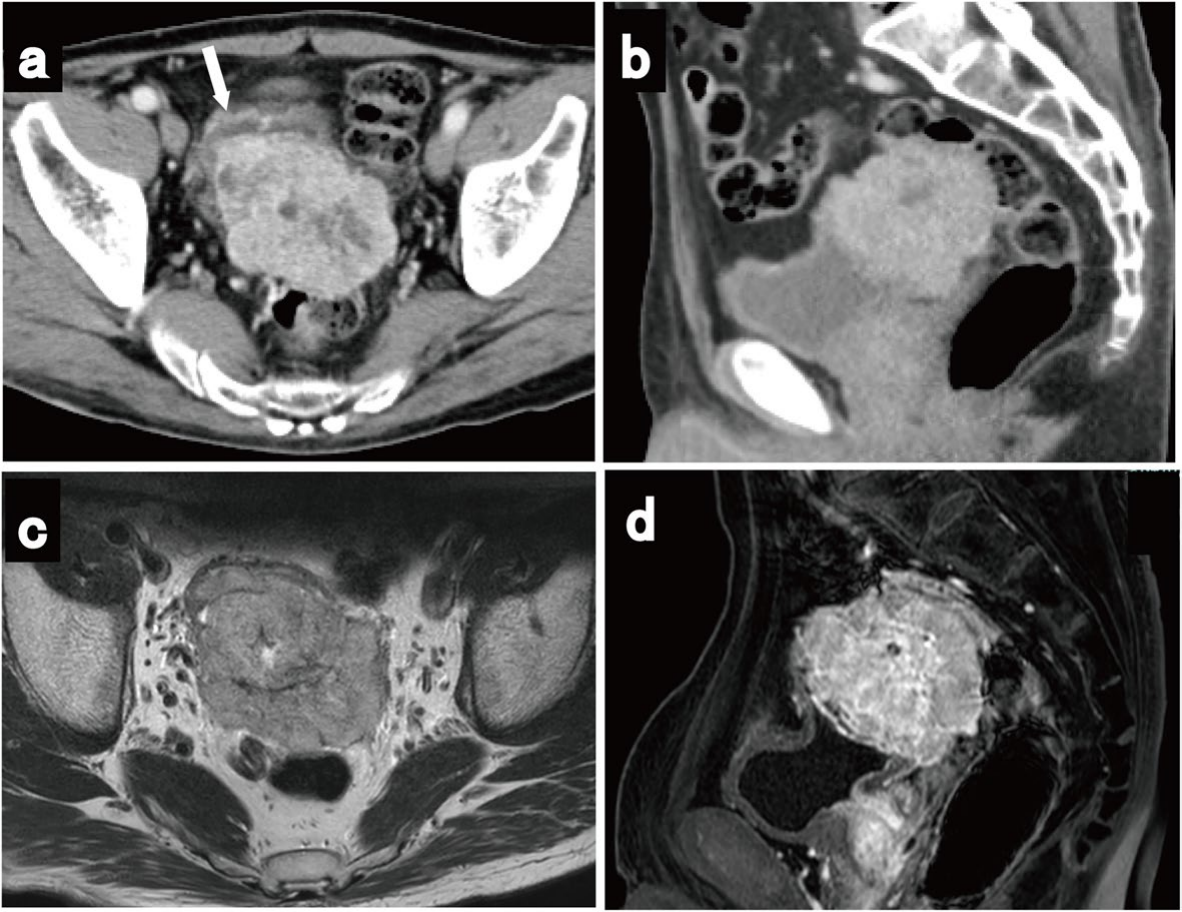
Contrast-enhanced computed tomography (CT), magnetic resonance imaging (MRI), and positron emission tomography/computed tomography findings. **a** Computed tomography (CT) revealing a tumor measuring 77 mm in diameter in the posterior wall of the urinary bladder (white arrow: urinary bladder), with heterogeneous enhancement in the solid part and an enhancing hypodense lesion. **b** Sagittal CT showing the tumor located between the posterior wall of the bladder and rectum. **c** MRI revealing a large tumor between the posterior wall of the bladder and rectum; the tumor is heterogeneous and partly hyperintense on a T2-weighted image. **d** Sagittal contrast-enhanced MRI showing an indistinct border between the tumor and posterior wall of the bladder, with the rectum compressed dorsally

**Fig. 2:**
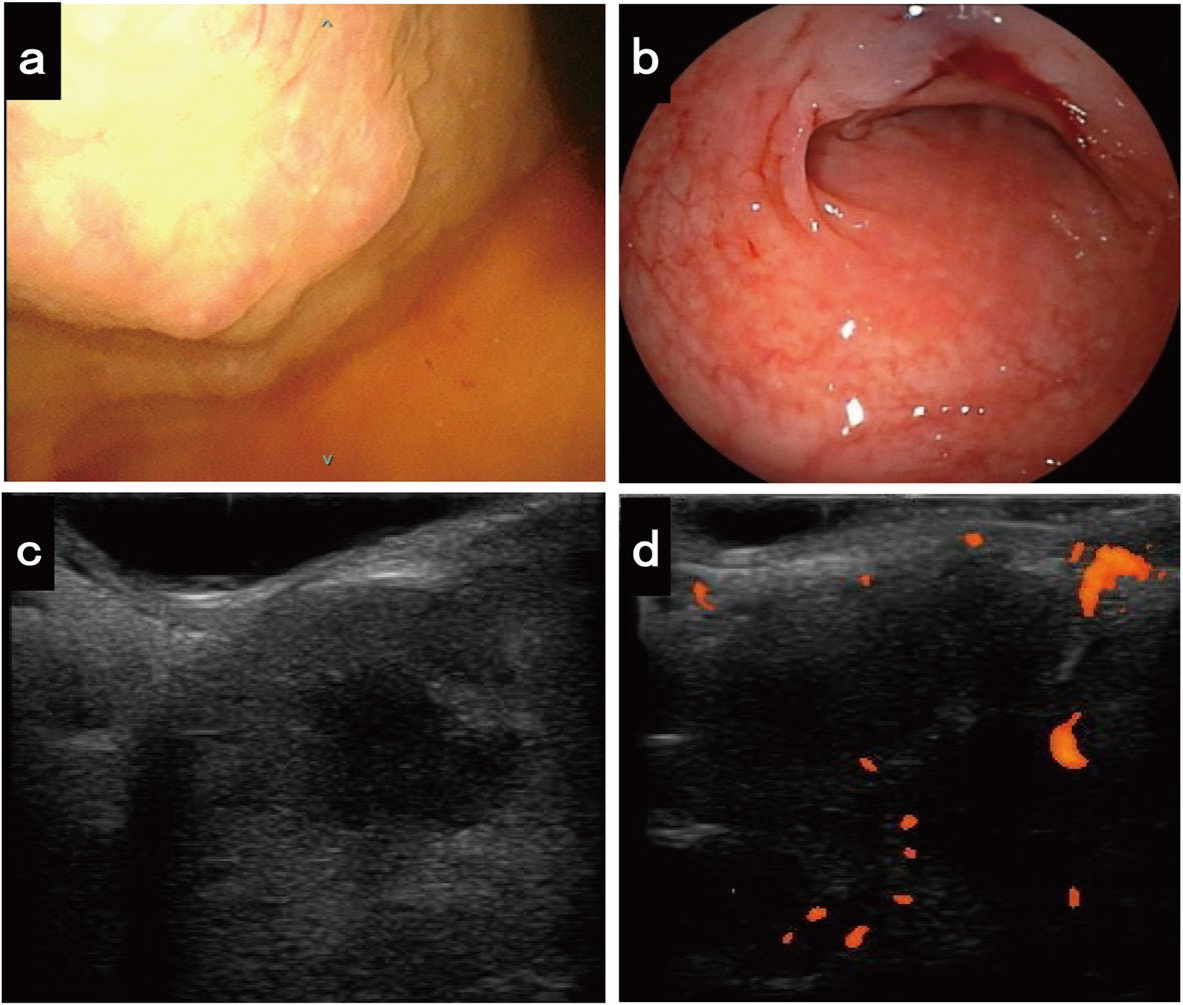
Findings of cystoscopy, colon fibroscopy, and endoscopic ultrasonography. **a** Cystoscopy showing compression of the trigone of the bladder by external masses, but no visible tumor in the lumen. **b** Colon fibroscopy showing compression of the anterior rectal wall and no tumor in the tract. **c** Endoscopic ultrasonography revealing clearly defined hypoechoic mass localized in the anterior wall of the rectum. **d** Doppler-echo showing that the mass is well vascularized at the periphery

Contrast-enhanced computed tomography (CT) showed a 77-mm diameter tumor in the posterior wall of the urinary bladder, with an unclear border with the rectum. The tumor showed heterogeneous enhancement in the solid part with an enhancing hypodense lesion (Fig. 1a and b), and no enlarged pelvic lymph nodes were observed. Magnetic resonance imaging (MRI) revealed a large tumor between the posterior wall of the bladder and the rectum, showing a low signal on T1-weighted imaging and a slightly high signal on T2-weighted imaging (Fig. 1c). Sagittal contrast-enhanced MRI showed an indistinct border between the tumor and posterior wall of the bladder, with the rectum compressed dorsally. (Fig. 1d). Moreover, 18F-fluorodeoxyglucose positron emission tomography (PET)-CT showed an intense hypermetabolic lesion in the tumor. Cystoscopy revealed external masses compressing the trigone of the bladder, but no tumor was visible in the lumen (Fig. 2a). Urine cytology revealed no tumor cells, and colon fibroscopy revealed compression of the anterior rectal wall and no tumor in the tract (Fig. 2b).

Based on the results of these examinations, a neoplasm of the pelvis of unknown origin was suspected. Therefore, we performed endoscopic ultrasonography fine-needle aspiration (EUS-FNA) biopsy of the rectal wall to diagnose the tumor. Endoscopic ultrasonography showed a large, well-vascularized, well-defined tumor, but the border with the rectum was partially obscure (Fig. 2 c and d). EUS-FNA of the tumor revealed spindle-shaped cells within fibrous tissue. Immunohistochemical staining was positive for CD34 but negative for S-100, c-kit, β-catenin, and desmin, suggesting a mesenchymal neoplasm such as a gastrointestinal stromal tumor (GIST) of the pelvis originating from the rectum. Preoperative neoadjuvant therapy using imatinib, for this case, was discussed during a multidisciplinary team meeting. However, the decision to operate was made because neoadjuvant therapy using imatinib for resectable GIST is still in clinical trials in Japan. Additionally, although the biopsy results were suggestive of GIST, they did not lead to a definitive diagnosis.

During laparotomy, the tumor was located in the posterior bladder wall and was suspected of having invaded the rectum. Therefore, we performed en bloc multivisceral resection (total cystectomy and low anterior resection of the rectum) to obtain a clear resection margin. During surgery, the patient’s systolic blood pressure abruptly increased to 190 mmHg when the tumor was touched. Perdipine stabilized the blood pressure; however, after removing the tumor, the blood pressure decreased, and catecholamine was required. After that, the patient had no further blood pressure attacks and an uneventful postoperative course.

**Fig. 3:**
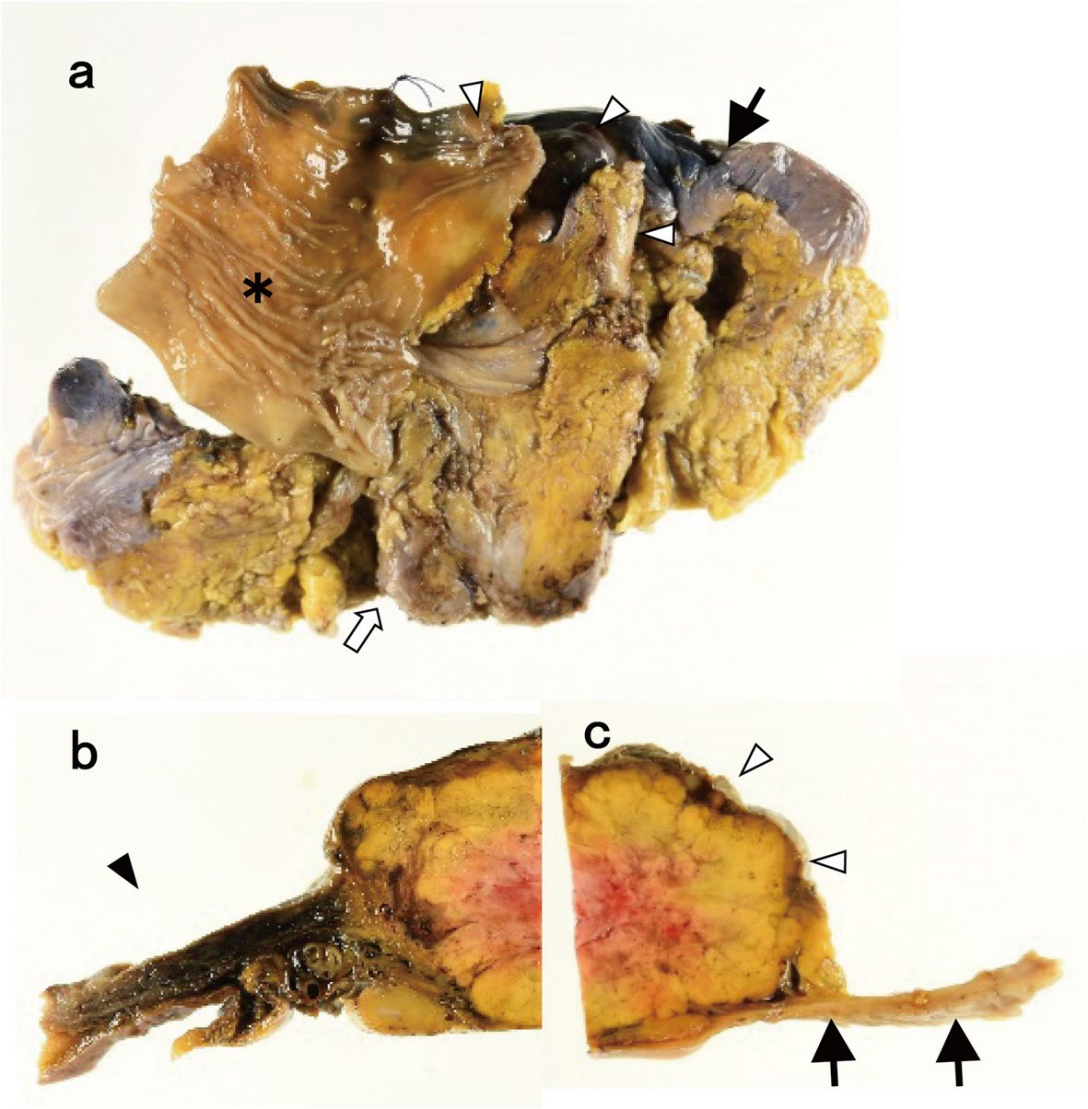
Histopathological analysis of the resected specimen involving the bladder, rectum, and prostate. **a** Gross appearance of the tumor. The tumor is surrounded by the bladder, rectum, and prostate. The tumor is 85 × 60 × 45 mm in size. (Black arrow, serosa side of the bladder; white arrowhead, tumor; asterisk, rectal mucosal surface; white arrow, prostate). **b** Gross appearance of the cut surface on the bladder side showing a well-circumscribed, yellowish-brown solid mass proliferating in the bladder muscularis and subserosal layers (black arrowhead: luminal side of the bladder). **c** Gross appearance of the cut surface on the rectal side. Inflammatory adhesions to the rectum are seen without any invasion (black arrow, luminal side of the rectum; white arrowhead, tumor)

**Fig. 4:**
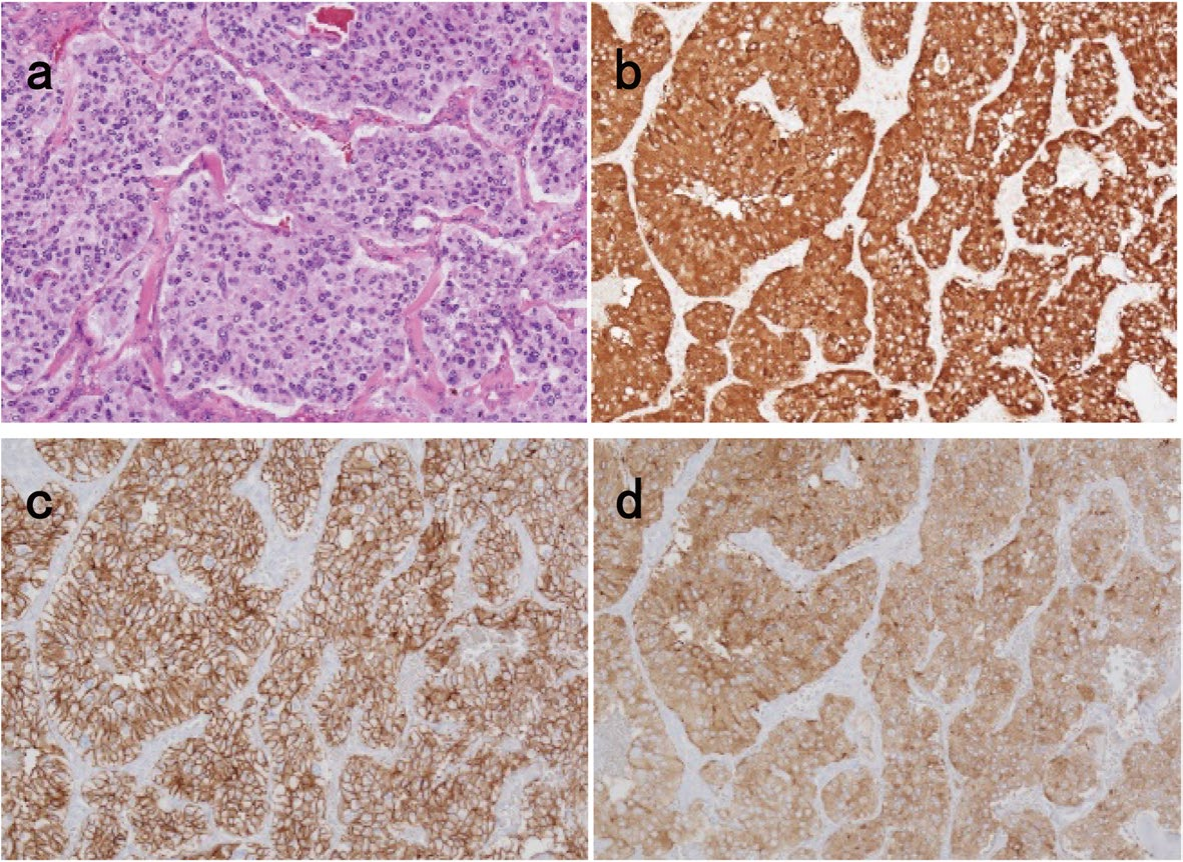
Pathological findings with histological and immunocytochemical staining. **a** The tumor, with the formation of nests and cords of cells delimited from each other by connective tissue and vascular septations (hematoxylin-eosin × 200). **b** Tumor cells positive for chromogranin A (× 200). **c** Tumor cells positive for CD56 (× 200). **d** Tumor cells positive for synaptophysin (× 200)

Macroscopic examination showed that the tumor measured 85 × 60 × 45 mm and was surrounded by the bladder, prostate, and rectum (Fig. 3a). The cut surface of the tumor revealed a well-circumscribed, yellowish-brown solid mass invading the muscularis and subserosal layers ofthe bladder (Fig. 3b), with no invasion of the rectal wall (Fig. 3c). Microscopic findings on hematoxylin-eosin staining revealed nests or cords of cells delimited by connective tissue and vascular septations, with a granular or clear amphophilic cytoplasm with an oval nucleus (Fig. 4a). Tumor cells proliferated from the muscular layer to the subserosa of the bladder and invaded the surrounding fatty tissue, but not the rectum. Immunohistochemistry was positive for chromogranin A, CD56, and synaptophysin (Fig. 4b, c, and d) and negative for keratin AE1/AE3, keratin CAM5.2, and SSTR2. Pub was confirmed by pathological and immunohistochemical examination. There was no recurrence at the 7-month follow-up.

## Discussion and conclusions

Neuroendocrine tumors arising from the adrenal medulla and extra-adrenal ganglia are called PCCs and PGLs, respectively [4]. Sympathetic PGL occurs along the prevertebral and paravertebral sympathetic chains and sympathetic nerve fibers innervating the retroperitoneum, thorax, and pelvis [1]. However, Pub is rare. Belian et al. [5] reviewed 106 cases of Pub in 80 articles. The mean patient age was 43.3 years (range: 11–84 years), the tumor affected both sexes equally (51 females and 55 males), and the mean tumor diameter was 3.9 cm (median: 3.45 cm, range: 1–9.1 cm) [5]. Endocrine tests can aid in Pub diagnosis because Pub was found to be hormone producing in 83% of cases [6]. Clinical symptoms of Pub are caused by catecholamine production and release [1], commonly hypertension (54.7%), headache (48.1%), hematuria (47.2%), and syncope and palpitations (43.4%) [5]. Micturition triggers headache, palpitations, sweating, and syncope (termed micturition attacks) in 52.8% of patients [5]. However, some cases lack common symptoms and present with abstract signs such as paresthesia and dyspnea; asymptomatic cases are rare [5]. In our case, the tumor was incidentally detected on CT. The patient had a history of hypertension, diabetes mellitus, and tachycardia-controlled long term with antihypertensives, pulse stabilizers, and antidiabetic medication. Thus, the patient did not exhibit symptoms that could be recognized as characteristic of Pub.

On cystoscopy, Pub is a hypervascularized, globular, submucosal mass protruding into the bladder with glossy or occasionally bleeding mucosa. CT demonstrates Pub as a well-defined, solid, vascular mass, or a cystic mass with foci of hemorrhage and necrosis located in the urinary bladder [7]. On T1- and T2-weighted MRI, Pub shows a hypo-to-isointense signal and a slight hyperintense signal, respectively, demonstrating strong enhancement [7–11]. However, CT and MRI findings are not specific to Pub, and GISTs have similar findings [12], which causes difficulty in distinguishing Pub from GISTs based on imaging findings.

So far, 11 cases of tumors larger than 8 cm have been reported, including this case (Table 1) [13–22]. Of these, seven had paroxysmal or persistent hypertension. Four cases with obvious catecholamine excess symptoms were preoperatively diagnosed by endocrine tests and without biopsy, and two were suspected to be rectal or prostatic tumors. Join et al. [13] reported a suspected prostate tumor based on imaging findings; however, biopsy results led to the diagnosis of Pub invading the prostate. In our case, although CT and MRI findings were consistent with Pub, the origin of the tumor was suspected to be rectal because it was located between the rectum and the bladder. Large tumors are occasionally difficult to diagnose on imaging because of invasion of the surrounding organs. Therefore, if a patient has a hypervascularized tumor in the pelvis, it is important to confirm the symptoms, especially if the patient has paroxysmal or persistent hypertension. Endocrine testing should be performed to rule out Pub.

**Table 1:** Previously reported cases of paraganglioma of the urinary bladder measuring > 8 cm

Reference	Year	Age (years)	Sex	Presenting symptoms	Size (cm)	Biopsy method	Preoperative diagnosis	Surgery
Lumb et al.	1958	56	Female	Hypertension, palpitations, headache, and diaphoresis with micturition	13	-	Pub	Tumor excision
Frydenberg et al.	1991	52	Male	Dysuria, hematuria, hypertension	8	TUR	Pub	Partial cystectomy
Kawai et al.	1993	47	Female	Hematuria, rheumarthritis	15	TUR	Bladder tumor	Partial cystectomy and hysterectomy
Naguib et al.	2002	21	Female	Headache, palpitations, anxiety, hypertension	9	-	Pub	Partial cystectomy
Ohta et al.	2010	44	Male	Hematuria	9.7	Percutaneous	Pub	Total cystectomy
Tsai et al.	2011	23	Male	Hematuria, dysuria, headache, and hypertension with micturition	9.1	TUR	Pub	Total cystectomy
Quist et al.	2015	58	Male	Hematuria	8	TUR	Bladder cancer	Total cystectomy
Sangwatanaroj et al.	2015	70	Female	Pulmonary edema, hypertension with micturition	8	-	Pub	Anterior pelvic exenteration
Havekes et al.	2015	42	Female	Hypertension	8	-	Pub	Partial cystectomy
Jain et al.	2016	16	Male	Hematuria, nausea, headache, sweating with micturition	8	TRUS	Pub	Total cystectomy
Our case	2021	64	Male	Hypertension, tachycardia	8.5	TRUS	GIST of the rectum	Total cystectomy with rectum resection

*TUR*, transurethral resection; *TRUS*, transrectal ultrasound

EUS is useful for evaluating and diagnosing intestinal submucosal and extraintestinal tumors and is occasionally performed for retroperitoneal PGLs and Pub [23–25]. In our case, EUS was performed to investigate tumor invasion into the rectum. It showed a heterogeneously hypoechoic, peripheral, and vascularized tumor on the anterior rectal wall. The tumor margins were largely well-defined; however, the border with the rectum was partially obscured, and the bladder wall was hidden due to the large tumor size. Therefore, we could not exclude the tumor invading the rectum. While cystoscopy of Pub and biopsy under EUS require caution due to the risk of bleeding and hypertensive crisis [24], biopsy for Pub can be an effective preoperative diagnostic method. Biopsy has low diagnostic accuracy for PGL and Pub [24], while that for EUS-FNA biopsy is 33% [23], and nondiagnostic or inconclusive biopsy findings are not rare [14–16, 26]. Conversely, Male et al. reported an 80% accuracy of transurethral biopsy using transurethral resection of bladder tumor (TURBT) for preoperative diagnosis of Pub in a study involving a small number of patients undergoing TURBT.

Preoperative diagnosis was not possible in those who did not undergo TURBT [27]. Pathological diagnosis via biopsy is difficult unless sufficient tissue is collected to recognize the characteristic pattern of PGL. Pub originates from chromaffin tissues and is mainly located in the muscular layer or lamina propria; thus, the biopsy sample must be collected from sufficiently deep tissues [27]. TURBT is considered more accurate than EUS-FNA biopsy because it allows tissue collection closer to the tumor. In our case, we could not collect enough tissue to correctly recognize the characteristic pattern of PGL, and rectal tissue was collected. Based on cystoscopy findings, the tumor was too large to be recognized as a submucosal tumor; therefore, a bladder tumor was not suspected, and TURBT was not performed. However, TURBT could have provided a pathologic biopsy diagnosis. TURBT should be considered for tumors that are difficult to recognize as submucosal in morphology but for which a transbladder biopsy approach is feasible.

Histologically, Pub is predominantly composed of chief cells arranged in cellular cords or cell nests (zellballen) surrounded by sustentacular cells and capillary networks [1, 4]. Neuroendocrine markers (synaptophysin and CD56) are almost always positive for PGL, and chromogranin A is always positive; CD34 is positive in Pub if capillaries are present [28, 29]. Positive reactions for catecholamine-synthesizing enzymes (dopamine beta-hydroxylase and tyrosine hydroxylase) help exclude other neuroendocrine tumors during differential diagnosis [1, 4]. Pub is rich in blood vessels within the tumor and neovascular proliferation around the tumor. Our resected specimen revealed typical characteristics of Pub. However, preoperative biopsy lacked the characteristic chief cells of Pub but showed spindle-shaped cells positive for CD34 antibody and negative for c-kit, s-100, and desmin expression; β-catenin did not show any reactivity, excluding desmoid positivity. The absence of reactivity for STAT6 and slight reactivity for CD34 eliminated the possibility of a solitary fibrous tumor. However, the possibility of KIT-negative GIST could not be excluded.

In many cases, diagnosis of Pub is made postoperatively. However, among the 38.9% of patients preoperatively diagnosed with Pub, micturition attacks allowed diagnosis in > 80% of cases [17, 30]. Conversely, preoperative diagnosis of Pub is difficult without characteristic symptoms. In our case, the following were the potential reasons for challenges in preoperative diagnosis: (1) the patient’s hypertension and tachycardia were controlled by medical treatment; thus, there were no typical symptoms; (2) the tumor was larger than a normal Pub, measuring 8.5 cm, with the ambiguous distinction of its contact with the rectum or bladder as the primary site; and (3) EUS findings showed an unclear border between the rectum and tumor; hence, the tumor was suspected to be of rectal origin. Therefore, in pelvic tumor cases, even large tumors, the possibility of Pub should be considered, with the performance of 123I-MIBG scintigraphy, and checks on the levels of catecholamines and their metabolites in blood and urine.

Hypertension must be stabilized 2 weeks before surgery using α-blocking agents or calcium channel blockers, and the tumor handled carefully, without exerting pressure, to inhibit the release of catecholamine. If peri-tumor manipulation causes blood pressure instability, the possibility of Pub should be considered early. The information must be shared between the anesthesiologist and surgeon to determine whether it is possible to continue the operation. Therefore, surgeons and anesthesiologists must be aware of the characteristics of Pub.

In conclusion, when a large hypervascularized tumor is found in the pelvis, Pub should be considered, and an endocrine examination should be performed, especially in patients with a history of hypertension or arrhythmia.

## Data Availability

Data sharing is not applicable to this article, as no datasets were generated or analyzed.

## Abbreviations

PCC: Pheochromocytoma
PGLs: Paragangliomas
Pub: Paraganglioma of the urinary bladder
CT: Computed tomography
MRI: Magnetic resonance imaging
PET: Positron emission tomography
EUS-FNA: Endoscopic ultrasonography fine-needle aspiration
GIST: Gastrointestinal stromal tumor
